# Leptin Induces a Novel Form of NMDA Receptor-Dependent LTP at Hippocampal Temporoammonic-CA1 Synapses^1^,^2^,^3^

**DOI:** 10.1523/ENEURO.0007-15.2015

**Published:** 2015-06-10

**Authors:** Xiao Luo, Gemma McGregor, Andrew J. Irving, Jenni Harvey

**Affiliations:** Division of Neuroscience, Medical Research Institute, Ninewells Hospital and Medical School, University of Dundee, Dundee DD1 9SY, United Kingdom

**Keywords:** excitatory synaptic transmission, leptin, NMDA receptor, synaptic plasticity

## Abstract

It is well documented that the hormone leptin regulates many central functions and that hippocampal CA1 pyramidal neurons are a key target for leptin action. Indeed, leptin modulates excitatory synaptic transmission and synaptic plasticity at the Schaffer-collateral input to CA1 neurons. However the impact of leptin on the direct temporoammonic (TA) input to CA1 neurons is not known. Here we show that leptin evokes a long-lasting increase [long-term potentiation (LTP)] in excitatory synaptic transmission at TA-CA1 synapses in rat juvenile hippocampus. Leptin-induced LTP was NMDA receptor-dependent and specifically involved the activation of GluN2B subunits. The signaling pathways underlying leptin-induced LTP involve the activation of phosphoinositide 3-kinase, but were independent of the ERK signaling cascade. Moreover, insertion of GluA2-lacking AMPA receptors was required for leptin-induced LTP as prior application of philanthotoxin prevented the effects of leptin. In addition, synaptic-induced LTP occluded the persistent increase in synaptic efficacy induced by leptin. In conclusion, these data indicate that leptin induces a novel form of NMDA receptor-dependent LTP at juvenile TA-CA1 synapses, which has important implications for the role of leptin in modulating hippocampal synaptic function in health and disease.

## Abstract (precis)

Hippocampal CA1 pyramidal neurons receive two anatomically distinct glutamatergic inputs that have distinct roles in learning and memory. The hormone leptin markedly influences excitatory synaptic transmission at the indirect Schaffer-collateral pathway to CA1 neurons.

## Significance Statement

Hippocampal CA1 pyramidal neurons receive two anatomically distinct glutamatergic inputs that have distinct roles in learning and memory. The hormone leptin markedly influences excitatory synaptic transmission at the indirect Schaffer-collateral pathway to CA1 neurons. However, the impact of leptin on the anatomically distinct temporoammonic (TA) input to CA1 pyramidal neurons is unknown. Here we provide the first compelling evidence that leptin induces at TA-CA1 synapses a novel form of NMDA receptor-dependent long-term potentiation (LTP) that shares similar expression mechanisms to activity-dependent LTP at this synapse. As the direct TA pathway is required for memory consolidation and place cell firing, these findings have important implications for leptin’s role in regulating spatial information and long-term memory consolidation.

## Introduction

It is well established that hippocampal CA1 pyramidal neurons receive two anatomically distinct glutamatergic inputs (van Strien et al., 2009). Distal dendritic regions within the stratum lacunosum-moleculare (SLM) are innervated directly by the perforant path, originating in layer III of the entorhinal cortex [known as the temporoammonic (TA) input]. Proximal regions of apical dendrites in stratum radiatum (SR) indirectly receive inputs from the CA3 region via the Schaffer-collateral (SC) pathway. The interplay between the dual inputs to CA1 pyramidal neurons is thought to be pivotal for processing, storing, and retrieving information in the hippocampus. Indeed, it has been shown that TA-CA1 synapses are strongly activated during cognitive tasks. In rodents, TA-CA1 synapses play a role in spatial learning in the Morris water maze (Nakashiba et al., 2008) and are also necessary for long-term memory consolidation (Remondes et al., 2004). There is also evidence that activity-dependent synaptic plasticity induced at TA-CA1 synapses can modulate synaptic plasticity at SC-CA1 synapses (Levy et al., 1998; Remondes and Schuman, 2002, 2004). Recent evidence has shown not only that there are distinct differences in the functional expression of receptors between TA-CA1 and SC-CA1 synapses, but also that synaptic activity induced by the two inputs is differentially regulated by neurotransmitters (Otmakhova and Lisman, 2000; Otmakhova et al., 2005).

It is well documented that the antiobesity hormone leptin regulates food intake and body weight via its hypothalamic actions (Spiegelman and Flier, 2001). However, the effects of leptin are not restricted to the hypothalamus as numerous studies indicate that leptin receptors are expressed throughout the brain and that leptin has widespread central actions (Harvey, 2007; Irving and Harvey, 2013). Indeed at hippocampal SC-CA1 synapses, leptin enhances NMDA receptor function and facilitates the induction of long-term potentiation (LTP; Shanley et al., 2001). In addition leptin promotes the induction of a novel form of *de novo* hippocampal long-term depression (LTD) under conditions of enhanced excitability (Durakoglugil et al., 2005). Recent evidence indicates that leptin increases the synaptic expression of GluA2-lacking AMPA receptors in adult hippocampus, resulting in a persistent increase in excitatory synaptic efficacy (Moult et al., 2010). Impairments in hippocampal synaptic plasticity and spatial memory have also been detected in leptin-insensitive rodents (*fa/fa* rats; *db/db* mice; (Li et al., 2002), whereas direct administration of leptin into the hippocampus improves memory processing in rodents (Wayner et al., 2004). However, the impact of leptin on TA-CA1 synapses is completely unknown.

Here we show that in contrast to SC-CA1 synapses, leptin induces a novel form of NMDA receptor-dependent LTP at juvenile hippocampal TA-CA1 synapses. Leptin-induced LTP was mediated by phosphoinositide (PI) 3-kinase-dependent signaling cascade and involved the synaptic insertion of GluA2-lacking AMPA receptors. Moreover, synaptic-induced LTP prevented the leptin-driven increase in synaptic efficacy at TA-CA1 synapses and vice versa.

## Materials and Methods

Hippocampal slices (350 µm) were prepared from postnatal day (P) 14–P22 old male, Sprague-Dawley rats. Animals were killed by cervical dislocation in accordance with the University of Dundee animal care committee’s regulations. Brains were rapidly removed and placed in ice-cold artificial CSF (aCSF; bubbled with 95% O_2_ and 5% CO_2_) containing the following (in mm): 124 NaCl, 3 KCl, 26 NaHCO_3_, 1.25 NaH_2_PO_4_, 2 CaCl_2_, 1 MgSO_4_, and 10 d-glucose. Once prepared, parasagittal slices were allowed to recover at room temperature in oxygenated aCSF for 1 h before use. Slices were transferred to a submerged chamber maintained at room temperature and perfused with aCSF. In all slices, the dentate gyrus and CA3 region were removed.

Because TA-CA1 synapses are electrotonically remote from CA1 cell somata, we used standard extracellular recordings of local field EPSPs (fEPSPs) to monitor excitatory synaptic transmission at TA-CA1 synapses. Recording pipettes contained aCSF (3–5 MΩ) and were placed in SLM to record TA-CA1 responses. In some experiments, recording pipettes were positioned in the SR to record the SC input to CA1 synapses. The direct TA pathway was stimulated at 0.033 Hz, using a stimulus intensity that evoked peak amplitude ∼50% of the maximum. Synaptic field potentials were low-pass filtered at 2 kHz and digitally sampled at 10 kHz.

The slope of the evoked fEPSPs was measured and expressed relative to the preconditioning baseline. Baseline responses were set to ∼50% of the maximal response. Data were monitored on-line and analyzed off-line using the WINltp program (Anderson and Collingridge, 2007). The degree of LTP was calculated 30-35 min after addition of leptin and expressed as a percentage of baseline ± SEM. All data are expressed as means ± SEM, and statistical analyses were performed using paired *t* test (two-tailed; 95% confidence interval) or repeated-measures ANOVA for comparison of means within subject or two-way ANOVA with Tukey’s post hoc test for comparisons between multiple groups. *P* < 0.05 was considered significant.

## Results

### Pharmacological discrimination of excitatory synaptic transmission at TA-CA1 and SC-CA1 synapses

**Figure 1:**
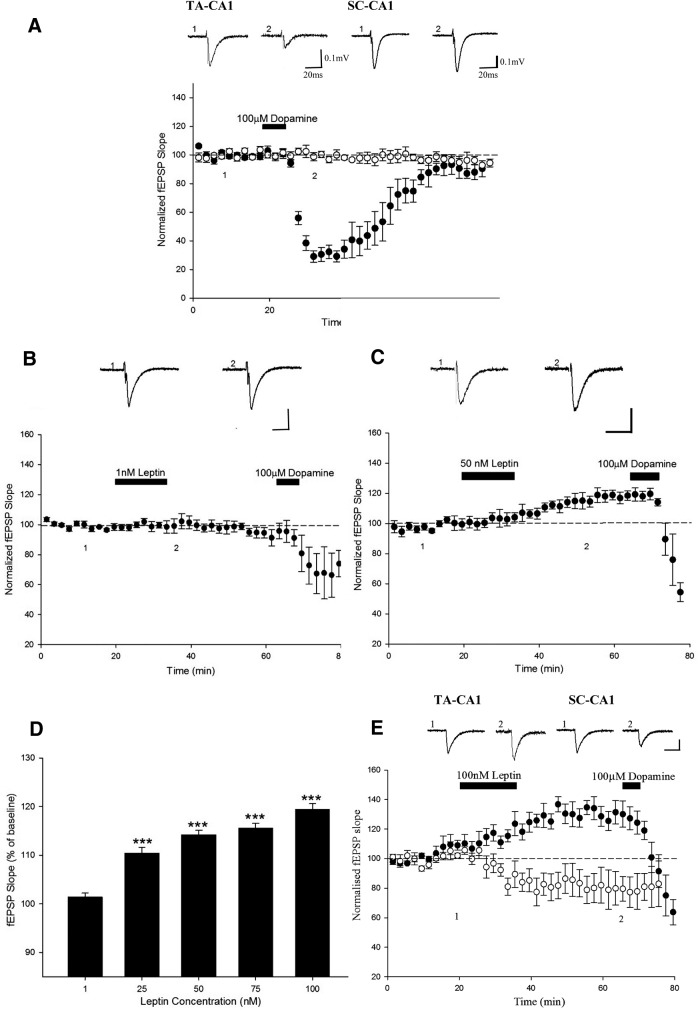
Leptin induces a persistent increase in excitatory synaptic transmission at TA-CA1 synapses. ***A,*** Plot of pooled and normalized data illustrating the effects of DA (100 μm) on excitatory synaptic transmission evoked at SC-CA1 (open circle) and TA-CA1 (filled circle) synapses in acute juvenile (P14–P21) hippocampal slices. Application of DA significantly attenuates synaptic transmission at TA-CA1, but not SC-CA1, synapses. ***B, C,*** Plot of pooled and normalized data illustrating the effects of 1 nm (***B***) and 50 nm (***C***) leptin on excitatory synaptic transmission evoked at TA-CA1 synapses. Application of 1 nm leptin had no effect on synaptic transmission, whereas 50 nm resulted in a persistent increase in excitatory synaptic transmission. In this (***A–C***) and subsequent figures, each point is the average of four consecutive fEPSP slope measurements, and representative synaptic traces for each experiment are shown above each plot and for the time indicated. Calibration: 0.1 mV, 50 ms. ***D,*** Histograms of pooled data illustrating the relative changes in synaptic transmission induced by different concentrations (1–100 nm) of leptin**. *E,*** Plot of pooled and normalized data obtained in two-input experiments that illustrate the simultaneous effects of leptin (100 μm) on excitatory synaptic transmission evoked at SC-CA1 (open circle) and TA-CA1 (filled circle) synapses in acute juvenile (P14–P21) hippocampal slices. Leptin induced opposing actions at the two inputs as synaptic transmission was enhanced at TA-CA1 synapses, but depressed at SC-CA1 synapses. Application of DA (100 mm) resulted in a significant depression of synaptic transmission at only TA-CA1 synapses. In this and subsequent figures *, **, and *** represent *P* < 0.05, *P* < 0.01, and *P* < 0.001, respectively.

Previous studies have demonstrated that application of high concentrations of dopamine (DA) result in a marked depression of excitatory synaptic transmission at TA-CA1 synapses, but have little effect on SC-CA1 synapses (Otmakhova and Lisman, 1999). The selective actions of DA at the TA-CA1 pathway is thought to play a role in filtering the excitatory drive onto pyramidal neurons. Thus, in order to ensure that we could discriminate between TA and SC inputs to CA1 neurons, we initially compared the effects of DA (100 µm) on excitatory synaptic transmission at TA and SC inputs to CA1 pyramidal neurons. In accordance with previous studies, application of DA (100 µm) for 5 min rapidly depressed excitatory synaptic transmission at TA-CA1 synapses to 31 ± 1.4% of baseline (*n* = 8; *P* < 0.001). In contrast, however, exposure to DA (100 µm) for 5 min had no significant effect on excitatory synaptic transmission at SC-CA1 synapses (98 ± 0.8% of baseline; *n* = 6; *P* > 0.05; Fig. 1*A*). Thus, in all subsequent experiments, DA (100 µm) was routinely applied at the end of experiments to verify recordings were from TA-CA1 synapses.

### Leptin evokes a concentration-dependent increase in excitatory synaptic transmission at TA-CA1 synapses

Previous studies indicate that leptin not only potently modulates excitatory synaptic transmission at SC-CA1 synapses, but that the direction of modulation is age and NMDA receptor subunit dependent (Moult and Harvey, 2011). Thus, at P11–P18 application of leptin results in transient synaptic depression that readily reverses on leptin washout (Moult and Harvey, 2011). In contrast leptin results in persistent increase in synaptic transmission in adult hippocampal slices that is sustained on washout of leptin (Moult et al., 2010; Moult and Harvey, 2011). Thus to examine the effects of leptin at TA-CA1 synapses, various concentrations of leptin were applied to acute hippocampal slices for 15 min. Application of 1 nm leptin had no effect on basal excitatory synaptic transmission (101 ± 0.9% of baseline; *n* = 5; *P* > 0.05; Fig. 1*B*). However, 25 nm leptin evoked a small transient increase in synaptic transmission (to 110 ± 0.9% of baseline; *n* = 8; *P* < 0.001) that returned to baseline levels (102 ± 0.6% of baseline) after 30 min washout of leptin (*n* = 4; *P* > 0.05). Application of higher concentrations of leptin (50–100 nm) also resulted in a significant increase in synaptic transmission, an effect that was sustained for ≥30 min after leptin washout (Fig. 1*C*). Thus synaptic transmission was increased to 118 ± 0.5% of baseline (*n* = 11; *P* < 0.001), 118 ± 2.2% of baseline (*n* = 4; *P* < 0.001), and 120 ± 0.4% of baseline (*n* = 23; *P* < 0.001) after treatment with 50, 75, and 100 nm leptin, respectively (Fig. 1*D*). These data indicate that leptin is capable of inducing a novel form of LTP of excitatory synaptic transmission at TA-CA1 synapses.

In order to further verify that leptin has opposing actions at the two inputs to CA1 neurons, and that DA is an appropriate pharmacological tool to discriminate between TA and SC pathways, two input experiments were performed. Thus, standard field potentials were recorded in response to alternate stimulation of the SC and TA pathways, and the effects of leptin (100 nm) and DA (100 µm) on both inputs were compared simultaneously. In agreement with previous studies (Shanley et al., 2001; Moult and Harvey, 2011), application of leptin resulted in a small depression of excitatory synaptic transmission (to 82 ± 10.2% of baseline) at SC-CA1 synapses (*n* = 6; *P* > 0.05), whereas a significant increase (to 131 ± 6.3% of baseline) in synaptic transmission was observed at TA-CA1 synapses (*n* = 6; *P* < 0.001; Fig. 1*E*). In addition, application of DA (100 µm) resulted in significant depression (∼56%) of synaptic transmission (from 131 ± 6.3% to 74 ± 2.3% of baseline; *n* = 6; *P* < 0.001) at TA-CA1 synapses, but failed to alter synaptic transmission at SC-CA1 synapses (from 82 ± 10.2% to 79 ± 2.4% of baseline; *n* = 6; *P* > 0.05). Thus these data confirm that leptin has opposing actions at the anatomically distinct inputs to CA1 neurons, but also that DA is an appropriate tool to discriminate between the distinct inputs.

### Leptin-induced LTP at TA-CA1 synapses has a postsynaptic locus of expression

**Figure 2:**
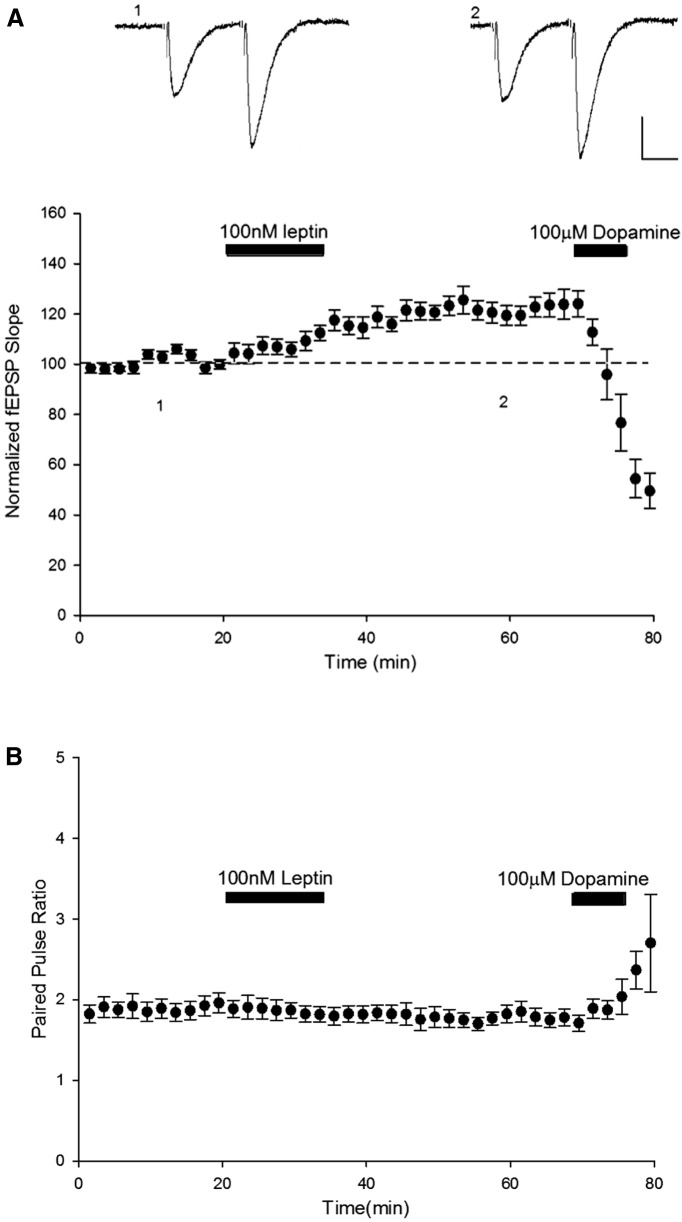
Leptin-induced LTP at TA-CA1 synapses involves a postsynaptic expression mechanism. ***A,*** Plot of pooled and normalized data illustrating the effects of 100 nm (15 min) leptin on excitatory synaptic transmission evoked at TA-CA1 synapses. Calibration: 0.1 mV, 20 ms. ***B,*** Corresponding plot of the pooled PPR against time for the experiments shown in ***A***. The effects of leptin on synaptic transmission were not accompanied by any significant change in PPR. In contrast, the synaptic depression induced by DA is accompanied by a significant alteration in PPR. Above the plots are representative pairs of fEPSPs evoked with a 50 ms interstimulus interval at the times indicated.

High levels of leptin receptor expression have been detected at both presynaptic and postsynaptic sites on hippocampal neurons (Shanley et al., 2002). Therefore leptin receptors located at either locus could mediate LTP induced by leptin at TA-CA1 synapses. Thus, to identify the locus of this effect of leptin at TA-CA1 synapses, we analyzed the paired-pulse facilitation ratio (PPR) during experiments by delivering two pulses at an interval of 50 ms. Changes in PPR classically reflect alterations in release probability (Pr). Under conditions where leptin increased excitatory synaptic efficacy (to 122 ± 1.3% of baseline; *n* = 9; *P* < 0.001) at TA-CA1 synapses, no significant change in PPR was detected during recordings (*n* = 9; *P* > 0.05; Fig. 2*A*,*B*), suggesting a postsynaptic expression mechanism. As a control, the effects of DA were also assessed as previous studies have shown that DA depresses excitatory synaptic transmission at TA-CA1 synapses via a presynaptic mechanism (Otmakhova and Lisman, 1999). In accordance with previous studies (Otmakhova and Lisman, 1999), application of DA (100 µm; 5 min) at the end of recordings resulted in significant depression of synaptic transmission, an effect that was accompanied by a significant increase in PPR from 1.82 ± 0.02 to 2.52 ± 0.13 (*n* = 9; *P* < 0.001; Fig. 2*A*,*B*). Thus, together these data indicate that leptin-induced LTP at TA-CA1 synapses is likely to involve a postsynaptic expression mechanism rather than altered Pr.

### Leptin-induced LTP at TA-CA1 synapses is NMDA receptor dependent

**Figure 3:**
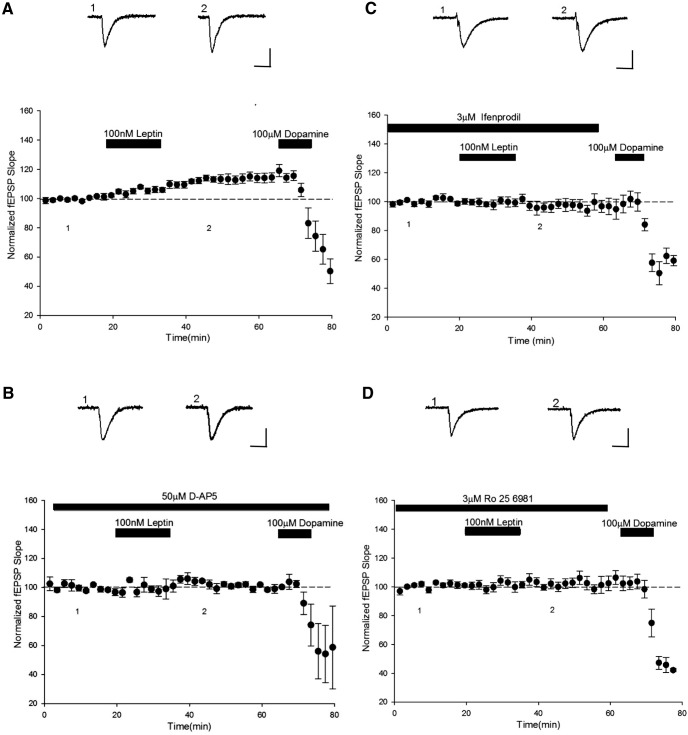
NMDA receptor activation is required for leptin-induced LTP at TA-CA1 synapses**. *A–D***, Plots of pooled and normalized data illustrating the effects of leptin (100 nm; 15 min) on TA-CA1 fEPSP slope in juvenile hippocampal slices. In control conditions (***A***) application of leptin resulted in LTP, whereas in the presence of d-AP5 (50 μm; ***B***), leptin failed to alter excitatory synaptic strength. ***C, D,*** Selective blockade of GluN2B subunits with either ifenprodil (3 µm; ***C***) or Ro 25-6081 (3 µm; ***D***) also prevented leptin-induced LTP. Calibration: 0.2 mV, 100 ms.

Previous studies have shown that NMDA receptor activation is pivotal for the induction of LTP at SC-CA1 synapses (Collingridge et al., 1983) and the synaptic insertion of AMPA receptors during hippocampal LTP (Man et al., 2003). The ability of leptin to induce LTP at adult hippocampal SC-CA1 synapses also requires activation of NMDA receptors (Moult et al., 2010). Furthermore, the ability of leptin to facilitate hippocampal LTP (Shanley et al., 2001), reverse established LTP (Moult et al., 2009), and induce a novel form of *de novo* LTD (Durakoglugil et al., 2005) are all NMDA receptor-dependent processes. Thus to verify whether the effects of leptin at TA-CA1 synapses involve NMDA receptors, the effects of the competitive NMDA receptor antagonist d-AP5 were assessed. Application of d-AP5 (50 µm; 30 min) had no effect on basal synaptic transmission per se (*n* = 4; *P* > 0.05). However, the ability of leptin to increase synaptic strength was significantly attenuated in the presence of d-AP5 such that leptin failed to increase synaptic transmission (100 ± 1.2% of baseline) in d-AP5-treated slices (*n* = 4; *P* > 0.05), an effect significantly different than that of interleaved control experiments (*n* = 4; *P* < 0.05; Fig. 3*A*,*B*). This suggests that an NMDA receptor-dependent process mediates leptin-induced LTP at TA-CA1 synapses.

### Activation of GluN2B-containing NMDA receptors is required for leptin-induced LTP

Several studies have shown that molecularly distinct NMDA receptors underlie different forms of activity-dependent synaptic plasticity in the hippocampus (Liu et al., 2004; Bartlett et al., 2007) and cortex (Massey et al., 2004). It is also known that subunit-specific alterations in the composition and localization of NMDA receptors occur during postnatal development (Monyer et al., 1994). Moreover, the subunit composition of NMDA receptors determines the polarity of leptin-induced synaptic plasticity at hippocampal SC-CA1 synapses (Moult and Harvey, 2011). Thus here we examined the role of GluN2B subunits in mediating the effects of leptin at TA-CA1 synapses. Application of two distinct GluN2B antagonists, ifenprodil (3 μm) or Ro-25 6981 (3 μm), had no effect on basal excitatory synaptic transmission per se at TA-CA1 synapses (*n* = 5 for each; *P* > 0.05). Exposure of hippocampal slices to leptin (100 nm) increased synaptic transmission to 115 ± 0.6% of baseline (*n* = 9; *P* < 0.001). In contrast, leptin failed to significantly alter synaptic transmission in the presence of either ifenprodil (99 ± 0.6% of baseline; *n* = 5; *P* > 0.05) or Ro-25 6981 (101 ± 0.8% of baseline; *n* = 4; *P* > 0.05; Fig. 3*C*,*D*). Together these data indicate that activation of GluN2B-containing NMDA receptors is required for leptin-induced LTP at juvenile TA-CA1 synapses.

### PI 3-kinase activation is required for leptin-induced LTP at TA-CA1 synapses

**Figure 4:**
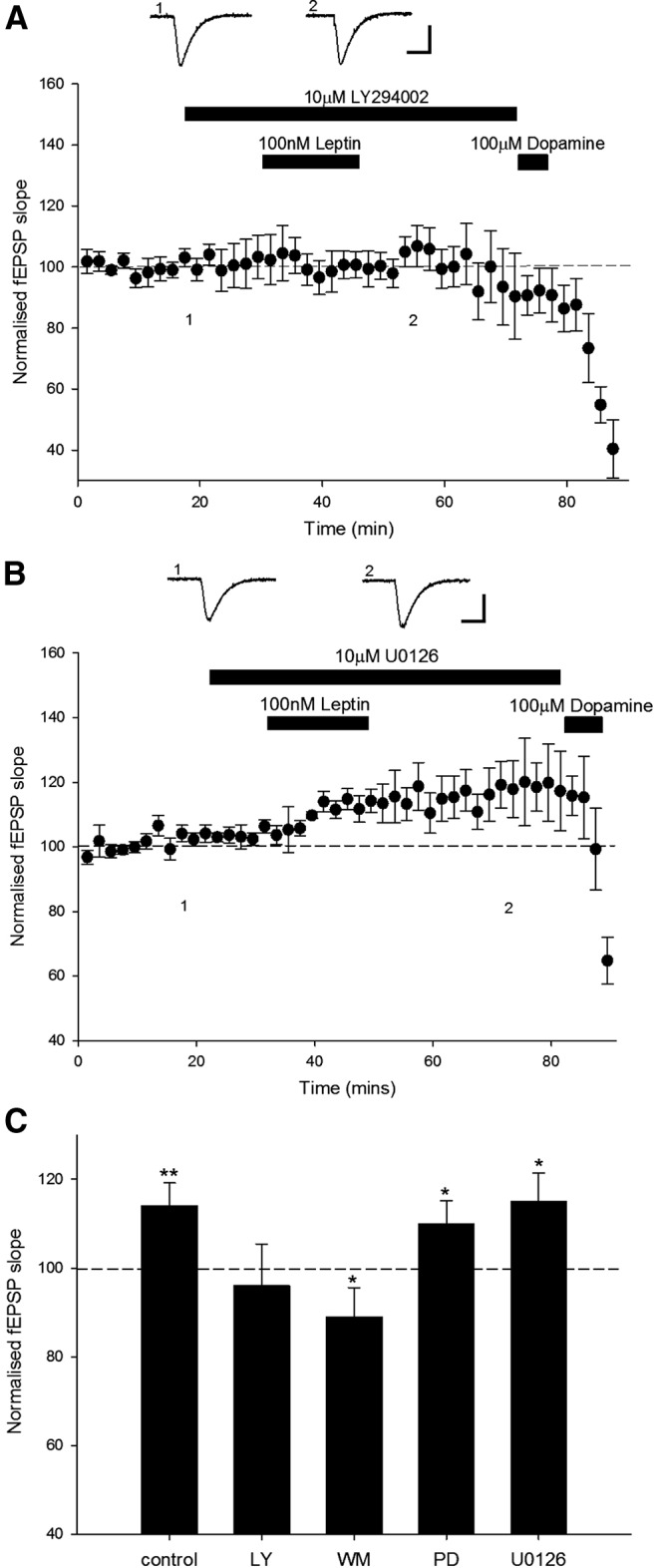
Leptin-induced LTP involves a PI 3-kinase-dependent process. ***A, B,*** Plots of pooled and normalized data illustrating the effects of leptin on synaptic transmission in the presence of the PI 3-kinase inhibitor LY294002 (10 μm; ***A***) or the ERK inhibitor U0126 (10 μm; ***B***), respectively. Leptin-induced LTP was prevented following blockade of PI 3-kinase, but not ERK. Calibration: 0.2 mV, 100 ms. ***C***, Histogram of the pooled data showing the relative effects of leptin (100 nm) on synaptic transmission in control conditions and in the presence of either LY294002 (10 μm), wortmannin (50 nm), U0126 (10 μm), or PD98059 (10 μm).

It is well documented that neuronal leptin receptor activation triggers a variety of downstream signaling cascades, including PI 3-kinase and ERK (Moult and Harvey, 2008). Indeed, activation of PI 3-kinase signaling underlies leptin-induced LTP at adult hippocampal SC-CA1 synapses (Moult, et al. 2010). Leptin-driven stimulation of the ERK signaling pathways has also been observed in hippocampal neurons (Shanley et al., 2001; O’Malley et al., 2007). Thus the persistent and transient synaptic depressions induced by leptin during early postnatal development involve the activation of the ERK, but not PI 3-kinase signaling pathway (Moult and Harvey, 2011). However, activation of both PI 3-kinase and ERK signaling contributes to leptin-induced facilitation of NMDA responses in hippocampal neurons (Shanley et al., 2001). Thus in the next series of experiments, the role of PI 3-kinase or MAPK signaling in leptin-induced LTP at TA-CA1 synapses was addressed. In order to examine the role of PI 3-kinase, two structurally unrelated inhibitors of this enzyme, namely wortmannin and LY294002, were used. Incubation of hippocampal slices with either LY294002 (10 µm) or wortmannin (50 nm) had no effect on synaptic transmission per se (*n* = 4 for each; *P* < 0.05). However, blockade of PI 3-kinase activity prevented the ability of leptin to induce LTP, such that leptin failed to increase synaptic transmission in hippocampal slices treated with either LY294002 (97 ± 9.5% of baseline; *n* = 5; *P* > 0.05; Fig. 4*A*,*C*) or wortmannin (89 ± 6.6% of baseline; *n* = 5; *P* < 0.05; Fig. 4*C*). In contrast, in parallel studies the ability of leptin to enhance excitatory synaptic strength was not affected by prior exposure to two distinct inhibitors of ERK activation (Fig. 4*B*,*C*). Thus, application of leptin increased synaptic transmission to 110 ± 5.2% of baseline (*n* = 5; *P* < 0.5) and 115 ± 6.5% of baseline (*n* = 5; *P* < 0.5) in slices exposed to PD98059 or U0126, respectively. Thus, together these data indicate that the ability of leptin to enhance excitatory synaptic strength at TA-CA1 synapses involves a PI 3-kinase-dependent, but not an ERK-dependent, mechanism.

### Leptin-induced LTP involves insertion of GluA2-lacking AMPA receptors

**Figure 5:**
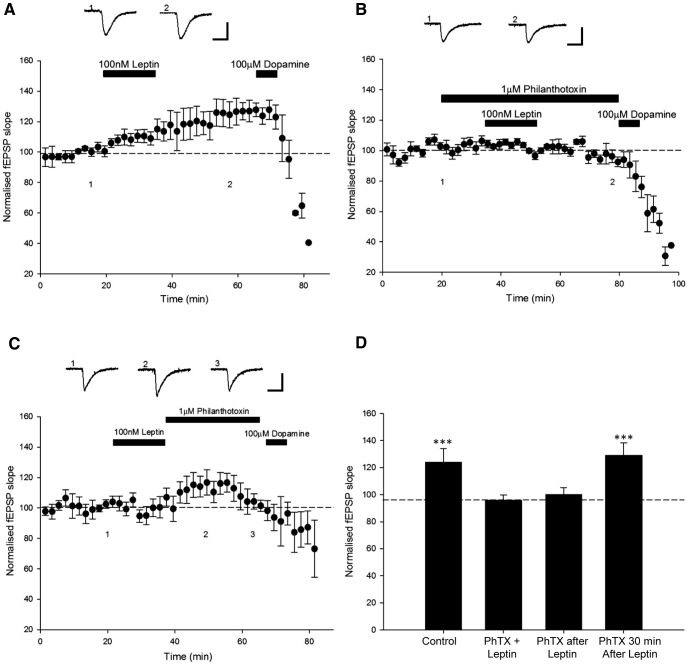
An increase in the synaptic density of GluR2-lacking AMPA receptors underlies leptin-induced LTP. ***A–C,*** Plots of pooled and normalized data illustrating the effects of leptin on synaptic transmission in control conditions (***A***), and in the presence of 1 μm philanthotoxin, the GluA2-lacking AMPA receptor inhibitor, applied either 15 min before (***B***) or immediately after (***C***) leptin addition. Prior treatment with philanthotoxin completely prevented leptin-induced LTP (***B***), whereas the leptin-driven increase in synaptic transmission was reversed by philanthotoxin. Calibration: 0.2 mV, 100 ms. ***D,*** Histogram of the pooled data showing the relative effects of leptin (100 nm) on synaptic transmission alone and in the presence of philanthotoxin (1 µm) applied before leptin application, immediately after leptin application, or 30 min after leptin washout.

It is well documented that trafficking of AMPA receptors to and away from synapses plays a key role in activity-dependent synaptic plasticity at hippocampal SC-CA1 synapses (Collingridge et al., 2004). The membrane insertion of AMPA receptors is also pivotal for hippocampal NMDA-dependent LTP (Man et al., 2003). The ability of leptin to induce LTP at adult hippocampal SC-CA1 synapses also involves trafficking of the AMPA receptor subunit GluA1 to synapses (Moult et al., 2010). Thus it is feasible that the ability of leptin to induce LTP at TA-CA1 synapses involves alterations in AMPA receptor trafficking processes. In order to examine this possibility, the effects of philanthotoxin, an inhibitor of GluA2-lacking AMPA receptors, was evaluated. Application of philanthotoxin (1 µm) to hippocampal slices had no effect on basal excitatory synaptic transmission (*n* = 4; *P* > 0.05). However, prior incubation of hippocampal slices with philanthotoxin (1 µm; 60 min) prevented the effects of leptin such that leptin failed to increase synaptic transmission (96 ± 3.9% of baseline; *n* = 5; *P* > 0.5; Fig. 5*A*,*B*) in philanthotoxin-treated slices. Moreover, application of philanthotoxin (1 μm) immediately after leptin addition reversed leptin-induced LTP as it resulted in a decrease in synaptic transmission from 115 ± 4.9% of baseline to 100 ± 5.3% (*n* = 5; *P* > 0.5; Fig. 5*C*). However, treatment with philanthotoxin 30 min after leptin washout failed to alter the magnitude of leptin-induced LTP (129 ± 9.2% of baseline; *n* = 6; *P* < 0.001; Fig. 5*D*). These data are consistent with an increase in the density of GluR2-lacking AMPA receptors underlying the induction and early maintenance phase of leptin-induced LTP at hippocampal TA-CA1 synapses.

### HFS-induced LTP at TA-CA1 synapses is also NMDA receptor dependent and expressed postsynaptically

**Figure 6:**
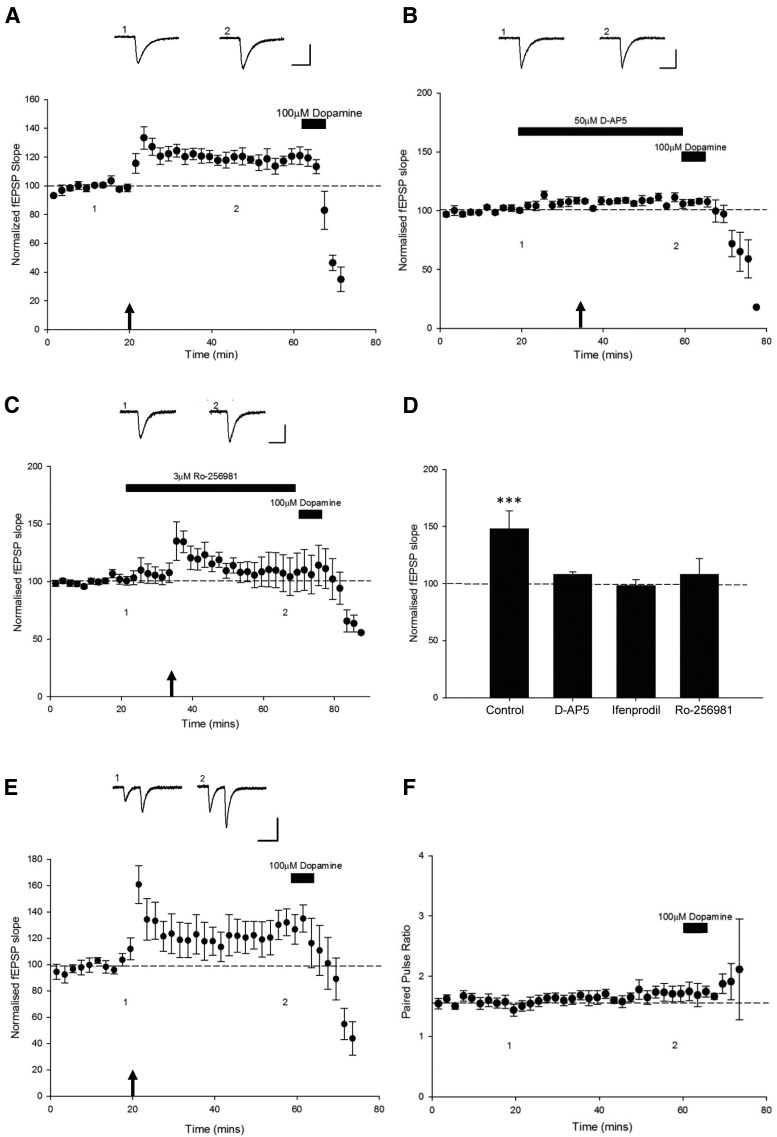
Activity-dependent synaptic plasticity at TA-CA1 synapses has a postsynaptic expression mechanism and is NMDA receptor dependent. ***A–C,*** Plots of pooled and normalized data illustrating the effects of the HFS paradigm (arrow) on excitatory synaptic transmission in control conditions (***A***), and in the presence of the competitive NMDA receptor antagonist d-AP5 (50 μm; ***B***) or the selective GluN2B antagonist Ro-256981 (3 μm; ***C***). Histograms of the pooled data showing the relative effects of the HFS paradigm on synaptic transmission in control conditions and in the presence of either d-AP5 (50 μm), ifenprodil (3 μm), or Ro-256981 (3 μm). ***E,*** Plot of pooled and normalized data illustrating the effects of HFS on excitatory synaptic transmission evoked at TA-CA1 synapses. ***F,*** Corresponding plot of the PPR against time for the experiments shown in ***E***. HFS-induced LTP at TA-CA1 synapses is not accompanied by any significant change in PPR. Calibration: 0.2 mV, 100 ms.

As leptin-induced LTP at TA-CA1 synapses displays parallels to classical activity-dependent LTP, the next series of experiments compared the properties of HFS-induced LTP at this synapse. In control slices, HFS (100 Hz, 1 s) resulted in a persistent increase in synaptic transmission to 148 ± 15.5% of control (*n* = 5; *P* < 0.001; Fig. 6*A*). It is well established that NMDA receptor activation is pivotal for activity-dependent synaptic plasticity at hippocampal SC-CA1 synapses (Collingridge et al., 1983). Previous studies have characterized the molecular mechanism underpinning long-lasting synaptic plasticity at TA-CA1 synapses and have identified a role for NMDA receptors in this process (Remondes and Schuman, 2003). Thus, in order to explore the role of NMDA receptors in HFS-induced LTP at TA-CA1 synapses, the effects of the competitive NMDA receptor antagonist d-AP5 were assessed. In contrast, in slices treated with d-AP5 (50 µm), the magnitude of LTP evoked by HFS was significantly attenuated to 108 ± 2.1% of control (*n* = 5; *P* > 0.05; Fig. 6*B*), indicating a key role for NMDA receptors in the induction of LTP at TA-CA1 synapses. As molecularly distinct NMDA receptors are implicated in different forms of activity-dependent synaptic plasticity (Liu et al., 2004; Bartlett et al., 2007), the specific role of GluN2B was examined using a selective inhibitor of GluN2B subunits, ifenprodil. In control slices, HFS resulted in LTP such that synaptic transmission increased to 116 ± 1.2% of baseline (*n* = 4; *P* < 0.001). However, in interleaved slices treated with ifenprodil (3 µm), HFS failed to induce LTP (98 ± 5.2% of baseline; *n* = 4; *P* > 0.05; Fig. 6*C*,*D*). Similarly, blockade of GluN2B subunits with Ro-25 6981 also prevented HFS-induced LTP (108 ± 13.8% of baseline; *n* = 5; *P* > 0.05; Fig. 6*D*). Thus these data indicate that HFS-induced LTP at TA-CA1 synapses is not only NMDA receptor dependent, but that activation of GluN2B-containing NMDA receptors plays a key role in this process.

In order to assess the locus of expression of HFS-induced LTP, the PPR was monitored during experiments by delivering two pulses at an interval of 50 ms. Under conditions where HFS induced LTP (to 121 ± 12.2% of baseline; *n* = 4; *P* < 0.05), no significant change in PPR was detected during recordings. Thus, the PPR was 1.5 ± 0.03 (*n* = 4) and 1.7 ± 0.02 (*n* = 4; *P* > 0.05; Fig. 6*E*,*F*) before and after HFS, respectively, suggesting a postsynaptic expression mechanism.

### ERK activation is required for HFS-induced LTP at TA-CA1 synapses

**Figure 7:**
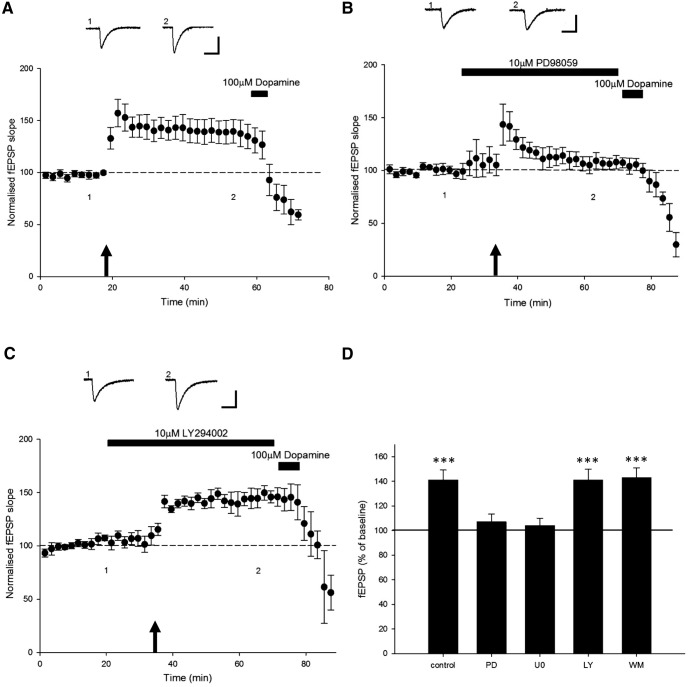
An ERK-signaling process underlies HFS-induced LTP at TA-CA1 synapses. ***A–C,*** Plots of pooled and normalized data illustrating the effects of the HFS paradigm (arrow) on excitatory synaptic transmission in control conditions (***A***) and in the presence of the ERK inhibitor PD98059 (10 mm; ***B***) or the PI 3-kinase inhibitor LY294002 (10 mm; ***C***). Calibration: 0.2 mV, 100 ms. ***D,*** Histograms of the pooled data showing the relative effects of the HFS paradigm on synaptic transmission in control conditions and in the presence of either PD98059 (10 μm), U0126 (10 μm), LY294002 (10 μm), or wortmannin (50 nm). Blockade or ERK, but not PI 3-kinase, activity prevented HFS-induced LTP.

We have shown that leptin-induced LTP at TA-C1 synapses is governed by PI 3-kinase signaling. Therefore, we next investigated whether this is also true for classical HFS-induced LTP at TA-CA1 synapses. Incubation of slices with inhibitors of ERK activation, namely PD98059 (10 µm; 55 min) or U0126 (10 µm; 55 min), had no effect on excitatory synaptic transmission per se (*n* = 4 for each). However, blockade of ERK signaling prevented the ability of HFS to induce LTP at TA-CA1 synapses such that there was no significant change in synaptic transmission with either PD98059 (107 ± 6.4% of baseline; *n* = 5; *P* > 0.5) or U0126 (104 ± 6.0% of baseline; *n* = 5; *P* > 0.5; Fig. 7*B*,*D*).

Conversely, in parallel studies, the magnitude of HFS-induced LTP was not affected after exposure to PI 3-kinase inhibitors. Hence, HFS induced LTP to 141 ± 9.0% of baseline (*n* = 5; *P* < 0.001) and 143 ± 8.0% of baseline (*n* = 5; *P* < 0.001) in slices exposed to either wortmannin (50 nm; 55 min) or LY294002 (10 µm; 55 min), respectively (Fig. 7*C*,*D*). Together, this indicates that HFS can induce a persistent enhancement in excitatory synaptic transmission at TA-CA1 synapses that requires the activation of an ERK-dependent but not a PI 3-kinase-dependent process. This contrasts with the involvement of PI 3-kinase in leptin-mediated LTP. Together these data suggest that leptin-induced LTP and HFS-induced LTP at the TA-CA1 synapse use distinct signaling pathways.

### GluR2-lacking AMPA receptors are required for the initial maintenance, but not the induction, of HFS-induced LTP at TA-CA1 synapses

**Figure 8:**
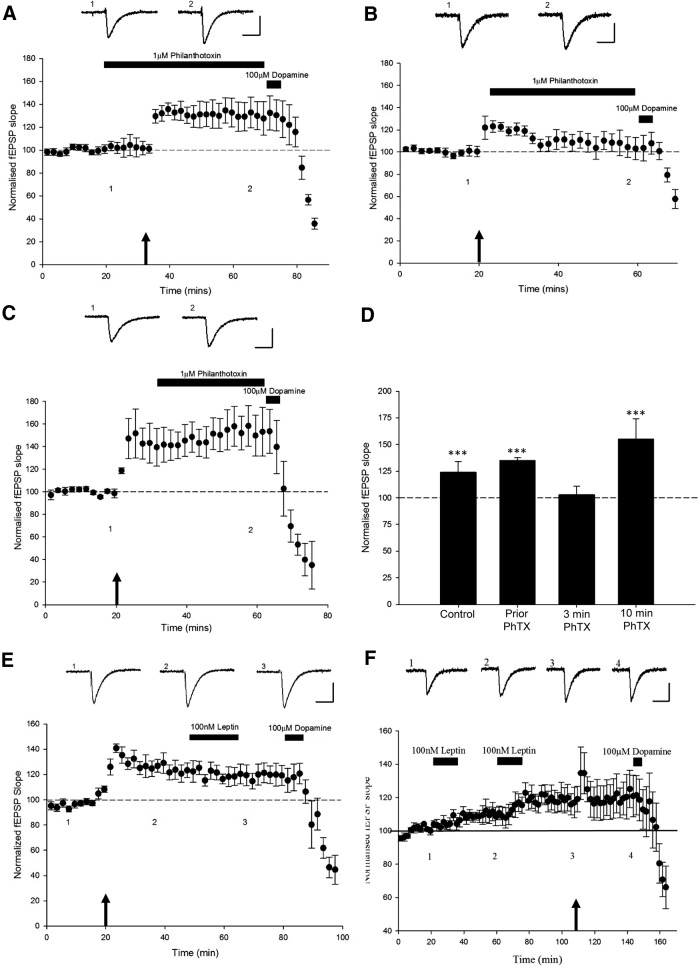
Leptin-induced LTP and activity-dependent LTP at TA-CA1 synapses share some similar expression mechanisms. ***A–C,*** Plots of pooled and normalized data illustrating the effects of the HFS paradigm (arrow) on excitatory synaptic transmission in slices exposed to philanthotoxin (1 mm) before HFS (***A***), 3 min after HFS (***B***), and 10 min after HFS (***C***). ***D,*** Histograms of the pooled data showing the relative effects of HFS on synaptic transmission in control conditions and following exposure to philanthotoxin before HFS, or 3 min or 10 min after HFS. LTP was reversed by philanthotoxin when applied 3 min after HFS, suggesting a role for insertion of GluA2-lacking AMPA receptors during the initial maintenance phase of LTP. ***E, F,*** Plots of pooled and normalized data illustrating the effects of HFS on excitatory synaptic transmission. ***E,*** Activity-dependent LTP occludes leptin-induced LTP. HFS resulted in a persistent increase in synaptic transmission that was unaffected by subsequent application of leptin. ***F,*** Leptin-induced LTP completely occludes activity-dependent LTP. Two consecutive applications of leptin resulted in increases in synaptic transmission. Subsequent HFS resulted in no further increase in excitatory synaptic strength. Calibration: 0.2 mV, 100 ms.

Our data indicate that leptin-induced LTP at TA-CA1 synapses requires an increase in the density of GluR2-lacking AMPA receptors. In order to compare whether HFS-induced LTP involves a similar expression mechanism, the effects of blocking GluA2-lacking AMPA receptors with philanthotoxin were addressed. Interestingly, application of philanthotoxin (1 µm; 55 min) before HFS did not inhibit the ability of this stimulation paradigm to induce LTP at TA-CA1 synapses (133 ± 2.5% of baseline; *n* = 5; *P* < 0.001; Fig. 8*A*,*D*). Furthermore, addition of philanthotoxin (1 µm; 30 min) 10 min after LTP induction also failed to inhibit the maintenance of LTP (155 ± 19.3% of baseline; *n* = 5; *P* < 0.001; Fig. 8*C*,*D*). However, when philanthotoxin (1 µm; 37 min) was applied 3 min after HFS induction, the effects were reversed and a decrease in synaptic transmission was observed to 103 ± 8% of baseline (*n* = 5; *P* > 0.5; Fig. 8*B*,*D*). This data implies that GluR2-lacking AMPA receptors may not be required for the induction of HFS-driven LTP but are critical for the initial maintenance phase of LTP.

### HFS-induced LTP and leptin-induced LTP at TA-CA1 synapses share similar expression mechanisms

As the long-lasting potentiation of synaptic transmission at TA-CA1 synapses induced by leptin displays a number of similarities to classical synaptically induced LTP, we assessed whether the two phenomena shared similar expression mechanisms by performing occlusion experiments. In the first series of experiments, HFS was applied initially to induce LTP. Then slices were treated with leptin 30 min after the HFS paradigm. HFS resulted in an increase in the magnitude of synaptic transmission to 122 ± 6.6% of baseline (*n* = 6; *P* < 0.001). Subsequent addition of leptin (100 nm) failed to increase synaptic transmission further (118 ± 8.1% of baseline; *n* = 6; *P* > 0.05), suggesting that classical activity-dependent LTP occludes leptin-induced LTP (Fig. 8*E*). In the next series of experiments, leptin was applied initially to induce LTP, and this was followed by further application of leptin to ensure saturation of leptin-induced LTP. This was then followed by delivery of HFS after 30 min washout of the second addition of leptin. Application of leptin (100 nm) resulted in a persistent increase in synaptic transmission to 110 ± 5.0% of baseline (*n* = 5; *P* < 0.01), whereas the second application of leptin resulted in a further increase to 118 ± 7.5% of baseline (*n* = 5; *P* < 0.001). Subsequent delivery of HFS failed to increase synaptic transmission further (120 ± 11.0% of baseline; *n* = 5; *P* < 0.05; Fig. 8*F*), an effect that was not significantly different to the magnitude of LTP induced by the second application of leptin (*P* > 0.05). Thus, these data indicate that leptin-induced LTP occludes activity-dependent LTP at TA-CA1 synapses.

## Discussion

The endocrine hormone leptin controls a number of central functions via its actions in the hypothalamus, including regulation of food intake and body weight (Spiegelman and Flier, 2001). However, numerous studies indicate that leptin targets many extrahypothalamic brain regions, including the hippocampus, where it is a potent regulator of excitatory synaptic function (Harvey, 2007; Irving and Harvey, 2013). Indeed, application of leptin to juvenile slices transiently reduces excitatory synaptic transmission at the SC input to hippocampal CA1 synapses (Shanley et al., 2001; Xu et al., 2008; Moult and Harvey, 2011). However, little is known about the impact of leptin on the anatomically distinct TA input to CA1 pyramidal neurons. Here we provide the first compelling evidence that, in contrast to the actions of leptin at classical SC-CA1 synapses, acute exposure to leptin induces a novel form of LTP at TA-CA1 synapses in juvenile (P14–P21) hippocampal slices. The increase in synaptic efficacy induced by leptin at TA-CA1 synapses is concentration dependent as high concentrations of leptin readily increased excitatory synaptic strength whereas low nanomolar concentrations were without effect. As previous studies have shown that hippocampal leptin receptors are distributed both presynaptically and postsynaptically (Shanley et al., 2002), the effects of leptin at TA-CA1 synapses may involve leptin receptors located at either site. However, in this study the persistent increase in synaptic efficacy induced by leptin was not associated with any significant alteration in the PPR, indicating that leptin-induced LTP likely involves a postsynaptic mechanism rather than changes in the probability of glutamate release. The involvement of a postsynaptic locus of expression in leptin-induced LTP at TA-CA1 synapses displays parallels to LTP induced by leptin at adult SC-CA1 synapses, which also involves a postsynaptic expression mechanism (Moult et al., 2010).

NMDA receptor activation is required for various effects of leptin on excitatory synaptic function at hippocampal SC-CA1 synapses, including leptin-induced facilitation and reversal of LTP (Shanley et al., 2001; Moult et al., 2009) as well as the induction of a novel form of LTD (Durakoglugil et al., 2005). Similarly in this study, the synaptic activation of NMDA receptors was pivotal for leptin-induced LTP as NMDA receptor blockade prevented the effects of leptin. It is known that the molecular composition of NMDA receptors varies during postnatal development, and that synaptic and extrasynaptic NMDA receptors are composed of distinct GluN2 subunits (Rumbaugh and Vicini, 1999). The distinct pattern of GluN2 expression in the forebrain is also functionally important as different GluN2 subunits are implicated in different forms of hippocampal activity-dependent synaptic plasticity (Liu et al., 2004; Bartlett et al., 2007). Here we show that activation of GluN2B-containing NMDA receptors underlies leptin-induced LTP at TA-CA1 synapses as treatment with ifenprodil or Ro 25-6981 to selectively inhibit GluN2B subunits blocked the effects of leptin. The involvement of GluN2B subunits in leptin-induced LTP in juvenile hippocampus correlates well with the higher levels of expression of GluN2B, rather than GluN2A, in the hippocampus at this stage of postnatal development (Monyer et al., 1994; Barria and Malinow, 2002). In contrast to the present study, however, leptin-induced LTP at adult SC-CA1 synapses involves activation of GluN2A, but not GluN2B, subunits (Moult and Harvey, 2011).

It is well established that two of the key signaling pathways activated downstream of hippocampal leptin receptors are PI 3-kinase and MAPK (ERK; Irving and Harvey, 2014). Moreover our recent studies indicate that there is divergence in the signaling pathways that couple leptin receptors to NMDA receptors at SC-CA1 synapses (Moult and Harvey, 2011). Thus, leptin-driven enhancement of GluN2B-mediated responses involves stimulation of the ERK signaling cascade at early postnatal stages. Conversely, in adult tissue, PI 3-kinase is implicated in the leptin-driven increase in GluN2A-mediated events (Moult and Harvey, 2011). In this study, activation of PI 3-kinase was key for leptin-induced LTP at TA-CA1 synapses as selective blockade of PI 3-kinase with either LY 294002 or wortmannin completely blocked the ability of leptin to enhance synaptic strength. Conversely, exposure to two distinct inhibitors of ERK activation, namely PD 98059 or U0126, failed to alter the magnitude of leptin-induced LTP. Thus, these data indicate that leptin-induced LTP at TA-CA1 synapses involves a PI 3-kinase-dependent process.

It is well documented that trafficking of AMPA receptors to and away from synapses is critical for activity-dependent synaptic plasticity (Collingridge et al., 2004). Moreover, LTP induction is reported to involve changes in density of GluA2 subunits at hippocampal SC-CA1 synapses (Plant et al., 2006). Alterations in AMPA receptor trafficking also play a key role in leptin-driven regulation of hippocampal synaptic function (Moult and Harvey, 2009). Indeed, insertion of the AMPA receptor subunit GluA1 into synapses underlies leptin-induced LTP at hippocampal SC-CA1 synapses (Moult et al., 2010). Similarly, leptin-induced LTP at TA-CA1 synapses involves altered AMPA receptor trafficking, as application of philanthoxin prior to leptin completely inhibited the ability of leptin to induce LTP. Blockade of GluA2-lacking AMPA receptors with philanthoxin also resulted in reversal of leptin-induced LTP when applied immediately after leptin. However, treatment of slices with philanthotoxin 30 min after leptin washout failed to reverse leptin-induced LTP, indicating that insertion of GluA2-lacking AMPA receptors is likely required for the initial induction phase, but not for the long-term maintenance of leptin-induced LTP.

Although the ability of leptin to induce LTP at TA-CA1 synapses contrasts with leptin action at classical SC-CA1 synapses in juvenile hippocampus, there are similarities to the LTP induced by leptin at adult SC-CA1 synapses (Moult et al., 2010). In particular, the ability of leptin to deliver GluA2-lacking AMPA receptors to TA-CA1 synapses is comparable to leptin regulation of AMPA receptor trafficking processes at adult SC-CA1 synapses. Indeed, our previous studies indicate that exposure to leptin increased the surface expression of GluA1, but not GluA2, subunits and also the synaptic density of GluA1 subunits in adult hippocampal slices (Moult et al., 2010). Moreover, leptin-driven inhibition of the phosphatase and tensin homolog and the resultant increase PI 3-kinase activity is critical for AMPA receptor insertion and leptin-induced LTP at adult SC-CA1 synapses (Moult et al., 2010). In accordance with this, LTP induced by leptin at juvenile TA-CA1 synapses also involves PI 3-kinase-dependent trafficking of GluA2-lacking AMPA receptors to hippocampal synapses. It is well documented that PI 3-kinase is a ubiquitous enzyme that phosphorylates PtdIns(4,5)P_2_, resulting in the generation of PtdIns(3,4,5)P_3_ (Cantley, 2002). Consequently as PI 3-kinase is implicated in leptin-induced LTP at TA-CA1 synapses, it is likely that an elevation in PtdIns(3,4,5)P_3_ levels also underlies the insertion on GluA2 subunits. In support of this possibility, the leptin-driven increase in PtdIns(3,4,5)P_3_ levels drives the synaptic insertion of GluA1 in hippocampal cultures (Moult et al., 2010). However, it is not clear at this stage how PI 3-kinase-dependent increases in the levels of PtdIns(3,4,5)P_3_ alters AMPA receptor trafficking. As the protein kinase Akt is a common target for PtdIns(3,4,5)P_3_, it is feasible that Akt is activated downstream of PI 3-kinase. In support of this possibility, Akt-dependent inhibition of glycogen synthase kinase-3 underlies the synaptic insertion of AMPA receptors following hippocampal LTP (Peineau et al., 2007).

Several studies indicate that leptin-dependent modification of excitatory synaptic strength share similar expression mechanisms with classical activity-dependent synaptic plasticity at hippocampal SC-CA1 synapses (Durakoglugil et al., 2005; Moult and Harvey, 2011). Thus it is possible that leptin-induced LTP also displays similarities with HFS-induced LTP at TA-CA1 synapses. In agreement with previous studies (Remondes and Schuman, 2002, 2003), application of an HFS paradigm resulted in robust LTP at TA-CA1 synapses in juvenile hippocampus. Moreover, the magnitude of HFS-induced LTP was comparable to the LTP induced by leptin at this synapse. In accordance with previous studies (Golding et al., 2002; Remondes and Schuman, 2003) and in line with many other forms of activity-dependent synaptic plasticity (Bliss and Collingridge, 1993), HFS-induced LTP required the synaptic activation of NMDA receptors as NMDA receptor blockade with d-AP5 prevented the induction of LTP. Moreover, in a manner similar to leptin-induced LTP, GluN2B-containing NMDA receptors play a role in HFS-induced LTP as selective inhibition of GluN2B subunits with either ifenprodil or Ro 25-6981 blocked HFS-induced LTP at TA-CA1 synapses. In this study, no significant change in PPR accompanied HFS-induced LTP, indicating that activity-dependent LTP at TA-CA1 synapses involves a postsynaptic expression mechanism.

Although both forms of synaptic plasticity are NMDA receptor dependent, our results suggest that divergent signaling cascades mediate leptin-induced LTP and HFS-induced LTP at TA-CA1 synapses. Indeed, in contrast to leptin-induced LTP, inhibition of ERK but not PI 3-kinase, blocked the induction of HFS-induced LTP at TA-CA1 synapses. However, there is overlap in the expression mechanisms that underlie both forms of synaptic plasticity as HFS-induced LTP occluded leptin-induced LTP, and there was occlusion of HFS-induced LTP when LTP induced by leptin was saturated after a second application of the hormone. In support of similar expression mechanisms, our data suggest that insertion of GluA2-lacking AMPA receptors plays a key role in both leptin-induced LTP and synaptically induced LTP. Indeed, application of the GluA2-lacking AMPA receptor inhibitor philanthotoxin 3 min after the HFS paradigm blocked LTP. In contrast, application of philanthotoxin before HFS failed to alter the magnitude of LTP, suggesting that insertion of GluA2-lacking AMPA receptors plays a role during the early maintenance phase, but not the induction phase, of synaptically induced LTP. Although our data indicate that the insertion of GluA2-lacking AMPA receptors is key for LTP at TA-CA1 synapses, the long-term maintenance of both forms of synaptic plasticity is unlikely to require GluA2-lacking AMPA receptors as philanthotoxin was without effect when applied either 10 min after HFS or 30 min after leptin.

Previous studies indicate that synaptic activity induced by the two inputs to CA1 neurons is differentially regulated by various neurotransmitters (Otmakhova et al., 2005). Here we show that there are also marked differences in the modulatory effects of the hormone leptin on the anatomically distinct SC and TA inputs to CA1 pyramidal neurons. At juvenile SC-CA1 synapses, leptin is reported to result in a synaptic depression (Moult and Harvey, 2011). In contrast, a novel form of LTP is induced by leptin at TA-CA1 synapses at the same stage of postnatal development. Indeed, the ability of leptin to bidirectionally modulate excitatory synaptic transmission at the different inputs to CA1 neurons was further verified in this study using two input experiments as opposing effects of leptin were observed. However the ability of leptin to modify excitatory synaptic transmission at both synaptic inputs to CA1 not only displays NMDA receptor dependence but also similar subunit-specific dependence as activation of GluN2B subunits was pivotal for the modulatory effects of leptin at both synaptic inputs. In contrast, however, divergent signaling cascades were found to underlie the modulatory effects of leptin at the two distinct pathways. Thus, activation of the ERK-dependent signaling mediates the transient synaptic depression induced by leptin at SC-CA1 synapses (Moult and Harvey, 2011), whereas leptin-induced LTP at TA-CA1 synapses requires the activation of PI 3-kinase.

Leptin circulates in the plasma and readily accesses the brain via transport across the blood–brain barrier. However, the concentrations of leptin in the brain may also arise from central sources as leptin mRNA and protein have been detected in several brain areas (Morash et al., 1999) and production of leptin within the brain has also been reported (Eikelis et al., 2007). Several studies have demonstrated that leptin is an important modulator of excitatory synaptic function at hippocampal SC-CA1 synapses (Harvey, 2007), a synaptic connection that plays a key role in mediating the cellular events underlying spatial learning and memory (Bliss and Collingridge, 1993). In contrast, the direct input to CA1 neurons from the entorhinal cortex (TA pathway) is implicated not only in long-term memory consolidation (Remondes and Schuman, 2002) but also in spatial representation and place cell maintenance (Brun et al., 2002; Ito and Schuman, 2007). Indeed, lesion studies have identified that the direct TA pathway is required for normal spatial firing in CA1 place cells (Brun et al., 2002, 2008). Thus the ability of leptin to modify excitatory synaptic strength at TA-CA1 synapses may have important implications for long-term memory consolidation and also in the regulation of place cell activity.

Several studies have found that rodents insensitive to leptin exhibit significant impairments in hippocampal synaptic plasticity and spatial learning and memory (Li et al., 2002; Gerges et al., 2003; Winocur et al., 2005), indicating that physiologically relevant concentrations of leptin are able to reach hippocampal synapses and modify synaptic function. It is well documented that cognitive deficits are linked to obesity-related diseases, such as type II diabetes (Gispen and Biessels, 2000), and that individuals with obesity and type II diabetes display neuronal resistance to leptin (Banks, 2004) even in the presence of high plasma leptin levels. Thus, it is likely that leptin resistance is a key factor that contributes to the development of impaired cognition in these CNS-driven diseases. In support of a link between obesity and cognitive deficits, patients with obesity and hypertension have lower cognitive function, whereas profound cognitive impairments and morbid obesity are common features in individuals with Prader–Willi syndrome (Gross-Tsur et al., 2001; Elias et al., 2003). Moreover, recent functional imaging studies have found that cerebral blood flow is significantly altered in specific brain regions in obese individuals (Matochik et al., 2005). In addition, a 5-year-old boy with congenital leptin deficiency treated with leptin displayed reinstatement of body weight to normal levels, but also significant improvement in his neurocognitive performance (Paz-Filho et al., 2008).

Recent evidence suggests a link exists between impaired leptin function and Alzheimer’s disease (AD; Beccano-Kelly and Harvey, 2012). Indeed, significant reductions in the circulating levels of leptin have been detected in individuals with AD (Power et al., 2001) and in rodents that model AD pathology (Farr et al., 2006). Furthermore, a number of studies have demonstrated protective actions of leptin against the neurotoxin amyloid beta (Aβ). Thus exposure of hippocampal slices to leptin prevents the detrimental effects of Aβ on activity-dependent synaptic plasticity at SC-CA1 synapses (Doherty et al., 2013). The entorhinal cortex, where the TA input originates, is also known to be an early target for degenerative brain disorders like AD. Indeed, phosphorylated tau accumulates in all layers of the entorhinal cortex prior to its buildup in the hippocampus (Braak and Braak, 1997). In addition, it is also known that the TA pathway synapses onto apical dendrites within the SLM, and in the early stages of AD phosphotau-positive dilatations occur on the apical dendrites in SLM, resulting ultimately in degeneration (Braak and Braak, 1997; Maurin et al., 2014). Thus, the ability of leptin to modify the strength of the TA input to CA1 neurons may be beneficial in preventing some of the deleterious early effects of phosphorylated tau on TA-CA1 synapses. In support of this possibility, recent evidence indicates that leptin regulates the levels of phosphorylated tau (Greco et al., 2010; Doherty et al., 2013).

It is known that excitatory synapses are altered in various models of depression and recent evidence indicates that glutamatergic dysfunction at TA-CA1 synapses is implicated in rodent models of chronic stress and depression (Kallarackal et al., 2013). Recent studies also suggest an antidepressant role for leptin as exposure of rodents to certain stress paradigms that model human depression results in a significant reduction in circulating leptin levels (Lu et al., 2006). Moreover, direct administration of leptin into the hippocampus mirrors the actions of antidepressant drugs in animal models of stress (Lu et al., 2006). Thus, the ability to modulate hippocampal TA-CA1 synapses may also have important implications for the antidepressant effects of leptin.

## Synthesis

The decision was a result of the Reviewing Editor Christian Luscher and the peer reviewers coming together and discussing their recommendations until a consensus was reached. A fact-based synthesis statement explaining their decision and outlining what is needed to prepare a revision is listed below. The following reviewers agreed to reveal their identity: Manuel Mameli, Camilla Bellone

The remaining issue that needs to be addressed before acceptance is the puzzling finding that leptin-LTP seems to depend on GluN2B containing NMDA receptors. There is general agreement, that at the age of the animals used here, these receptors are no longer present. Please discuss this issue and highlight the contradiction. Or better, carry out experiments to directly address this point.

Dear Professor Luscher,

We would be grateful if you would consider our amended manuscript for publication in eNeuro. We would like to thank the reviewers for their constructive general comments on our manuscript. Below is our response the individual point raised.

The remaining issue that needs to be addressed before acceptance is the puzzling finding that leptin-LTP seems to depend on GluN2B containing NMDA receptors. There is general agreement, that at the age of the animals used here, these receptors are no longer present. Please discuss this issue and highlight the contradiction. Or better, carry out experiments to directly address this point.

The reviewers have raised a point regarding the levels of expression of the GluN2B subunit at the stage of postnatal development that was tested in this study. In contrast to the reviewers' suggestion we disagree with the proposal that GluN2B is not expressed at all at P14-22 as it is well documented in the literature that there is significant expression of this particular subunit at this developmental stage. Indeed the original in situ hybridisation studies carried out by Monyer et al (1994; Neuron 12, 529-540) demonstrated very clearly high levels of GluN2B mRNA expressed prior to P14, and although this declined with age, significant levels of GluN2B were still clearly evident in adult hippocampus. In agreement with these studies, a number of subsequent studies have assessed the synaptic levels of GluN2B expressed on CA1 pyramidal neurons using electrophysiological approaches. Indeed, Bellone and Nicoll (2007, Neuron 55, 779-785) and Bartlett et al 2007 (Neuropharmacol, 52, 60-70) both explored the contribution of GluN2B subunits to NMDAR-mediated EPSCs using the selective GluN2B antagonists ifenprodil and Ro25 6981. Bellone and Nicoll clearly demonstrated that application of ifenprodil resulted in a significant reduction in the NMDA EPSC at P16-21, indicating expression of GluN2B on CA1 pyramidal neurons at this developmental stage. This finding was also replicated in the experiments performed by Bartlett et al, (2007), as pharmacological approaches also demonstrated that application of Ro25 6981 inhibited NMDA EPSCs in hippocampal slices at P14. In addition, recent studies from our laboratory have also examined the contribution of GluN2B subunits to synaptic NMDA EPSCs in CA1 neurons at different stages of postnatal development (Moult and Harvey, 2011, Neuropharmacol 61; 924-36). In accordance with previous studies we also found that ifenprodil significantly reduced the amplitude of NMDA EPSCs at P11-18, indicating significant expression of GluN2B subunits at a similar stage of postnatal development as the current study. Moreover, we also found significant levels of GluN2B subunits in adult hippocampus, as significant reductions in NMDA EPSCs were observed in the presence of ifenprodil in hippocampal slices from 3-4 month old animals. Thus, there is significant evidence in the literature that the GluN2B subunit is expressed on CA1 pyramidal neurons at the stage of postnatal development examined in the current study. In view of this, we feel there is no need for us to perform additional studies to verify this, or to justify our findings further in the manuscript.
